# Raman Spectroscopy for Rapid Evaluation of Surgical Margins during Breast Cancer Lumpectomy

**DOI:** 10.1038/s41598-019-51112-0

**Published:** 2019-10-10

**Authors:** Willie C. Zúñiga, Veronica Jones, Sarah M. Anderson, Alex Echevarria, Nathaniel L. Miller, Connor Stashko, Daniel Schmolze, Philip D. Cha, Ragini Kothari, Yuman Fong, Michael C. Storrie-Lombardi

**Affiliations:** 1 Harvey Mudd College, Department of Physics, 301 Platt Blvd., Claremont, CA 91711 USA; 2 City of Hope National Medical Center, Department of Surgery, 1500 E. Duarte Rd, Duarte, CA 91010 USA; 3 Harvey Mudd College, Department of Engineering, 301 Platt Blvd., Claremont, CA 91711 USA; 4 Kinohi Institute, Inc., 530S. Lake Avenue, Pasadena, CA 91101 USA

**Keywords:** Biophysics, Biotechnology

## Abstract

Failure to precisely distinguish malignant from healthy tissue has severe implications for breast cancer surgical outcomes. Clinical prognoses depend on precisely distinguishing healthy from malignant tissue during surgery. Laser Raman spectroscopy (LRS) has been previously shown to differentiate benign from malignant tissue in real time. However, the cost, assembly effort, and technical expertise needed for construction and implementation of the technique have prohibited widespread adoption. Recently, Raman spectrometers have been developed for non-medical uses and have become commercially available and affordable. Here we demonstrate that this current generation of Raman spectrometers can readily identify cancer in breast surgical specimens. We evaluated two commercially available, portable, near-infrared Raman systems operating at excitation wavelengths of either 785 nm or 1064 nm, collecting a total of 164 Raman spectra from cancerous, benign, and transitional regions of resected breast tissue from six patients undergoing mastectomy. The spectra were classified using standard multivariate statistical techniques. We identified a minimal set of spectral bands sufficient to reliably distinguish between healthy and malignant tissue using either the 1064 nm or 785 nm system. Our results indicate that current generation Raman spectrometers can be used as a rapid diagnostic technique distinguishing benign from malignant tissue during surgery.

## Introduction

Breast cancer is the leading cause of cancer death among females worldwide, accounting for 25% of all cancer cases and 15% of all cancer deaths^1–3^. While improvements in screening have enabled the early diagnoses of many breast cancers, the significant number of diagnoses that eventually lead to death (~20% at 15 years) provide the primary impetus for advances in surgical intervention^4,5^.

Complete excision of tumors represents a potentially curative option for treatment. However, in >30% of breast tumor excisions, the surgeon inadvertently cuts into tumor tissue and leaves cancer behind^6^. These positive surgical margins following lumpectomy are well-documented risk factors for local recurrence and disease-specific mortality. For invasive breast cancer, a positive margin is defined as tumor touching the inked margin^7^, and is typically discovered during postoperative microscopic pathologic assessment. Unfortunately, pathologic assessment of margins may take 1–2 weeks, is resource-intensive, and requires support from both a pathologist and a well-funded laboratory^8,9^. The belated finding of positive margins requires secondary surgery, potential surgical complications, patient discomfort, and financial burden for both the patient and the operating institution^10^.

Raman spectroscopy has emerged as a promising biochemical technique for real-time*, in vivo*, non-destructive detection of many types of cancer^11–15^. Raman generates biochemical fingerprints reflecting a tissue’s current biological composition and activity^16,17^. Multiple groups have demonstrated that healthy and malignant breast tissue produce distinct Raman spectra^18–20^. These differences are attributed to biochemical composition alterations in malignant tissue relative to healthy tissue, such as a reduced fatty-acid concentration, variable collagen content, and increases in spectral signatures associated with elevated concentrations of DNA, RNA, and peri-nuclear proteins in tumor sites when compared to healthy tissue^21,22^. Although previous generations of Raman spectroscopy systems have successfully detected specific biochemical signatures of cancer, our experience is that they have been too expensive, fragile, and/or cumbersome to deploy into widespread clinical use.

An inexpensive, portable Raman system capable of surveying cancer margins during initial surgery in real-time is desirable in both first and third world settings^23,24^. Fortunately, the use of Raman techniques in multiple disciplines has prompted the development of increasingly inexpensive commercial Raman systems and hand-held probes capable of safely interrogating biological targets^25–29^. Here we show that relatively inexpensive, off-the-shelf infrared Raman devices can be used to differentiate between malignant and healthy regions in resected breast tissue with a high degree of certainty.

## Results

### Raman spectra distinguish healthy and neoplastic tissue with both 1064 and 785 nm excitation

**Figure 1:**
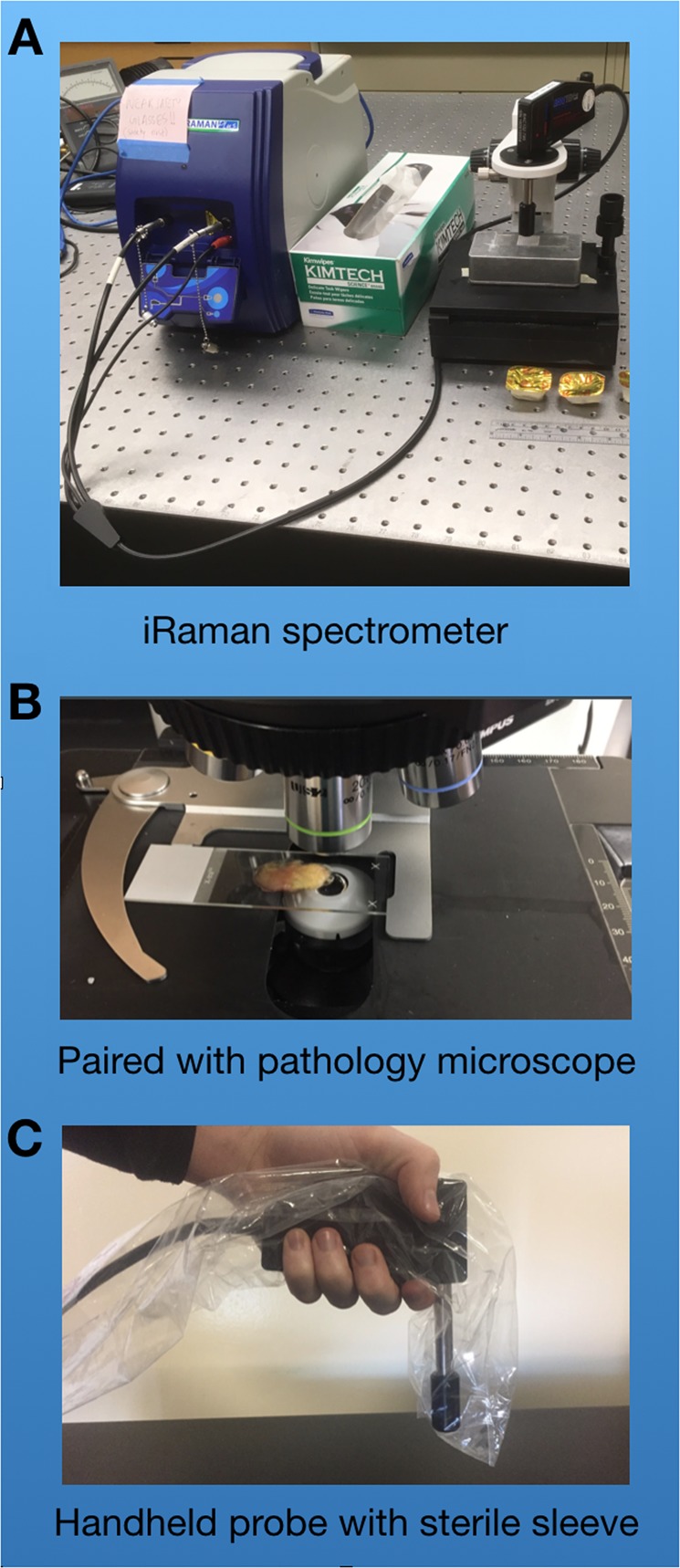
Figure 1A shows the housing and Raman probe head common to both the i-Raman Plus (785 nm) and i-Raman Ex (1064 nm) systems. The housing measures 6.7″ × 13.4″ × 9.2” (17 cm × 34 cm × 23.4 cm), weighs ~10 lbs (4.6 kg) and is designed for operating temperatures between 10oC and 35oC. Figure 1B shows the collection of data from a surgical specimen in microscope mode with the Raman probe head integrated into the optical axis of a standard laboratory microscope. Figure 1C shows the Raman probe head in hand held mode encased in a sterile surgical sleeve.

We evaluated two commercial Raman systems. Both operate in the infrared, one using a 1024 nm laser excitation source and the other 785 nm. Both wavelengths are known to be capable of interrogating biological systems without target damage. The 1064 nm systems probe more deeply into tissue than 785 nm devices and often generate significantly less fluorescence. That is a significant advantage since fluorescence can easily mask the weaker Raman signal. Unfortunately, systems operating at 1064 nm are significantly more expensive and usually exhibit a more limited spectral bandwidth and diminished spectral resolution. The two systems evaluated were the *i-Raman Ex 1064 nm* and *i-Raman Plus 785 nm*, both manufactured and distributed commercially by B&W Tek (Newark, DE). Both systems can be operated in microscopic or hand-held probe modes (see Fig. 1 and Methods). For initial evaluation, we employed the systems in microscopic mode and selected laser exposure times so that total laser exposure (laser excitation power x collection time) would equal 9 × 10^3^ mW-seconds for both systems.

**Figure 2:**
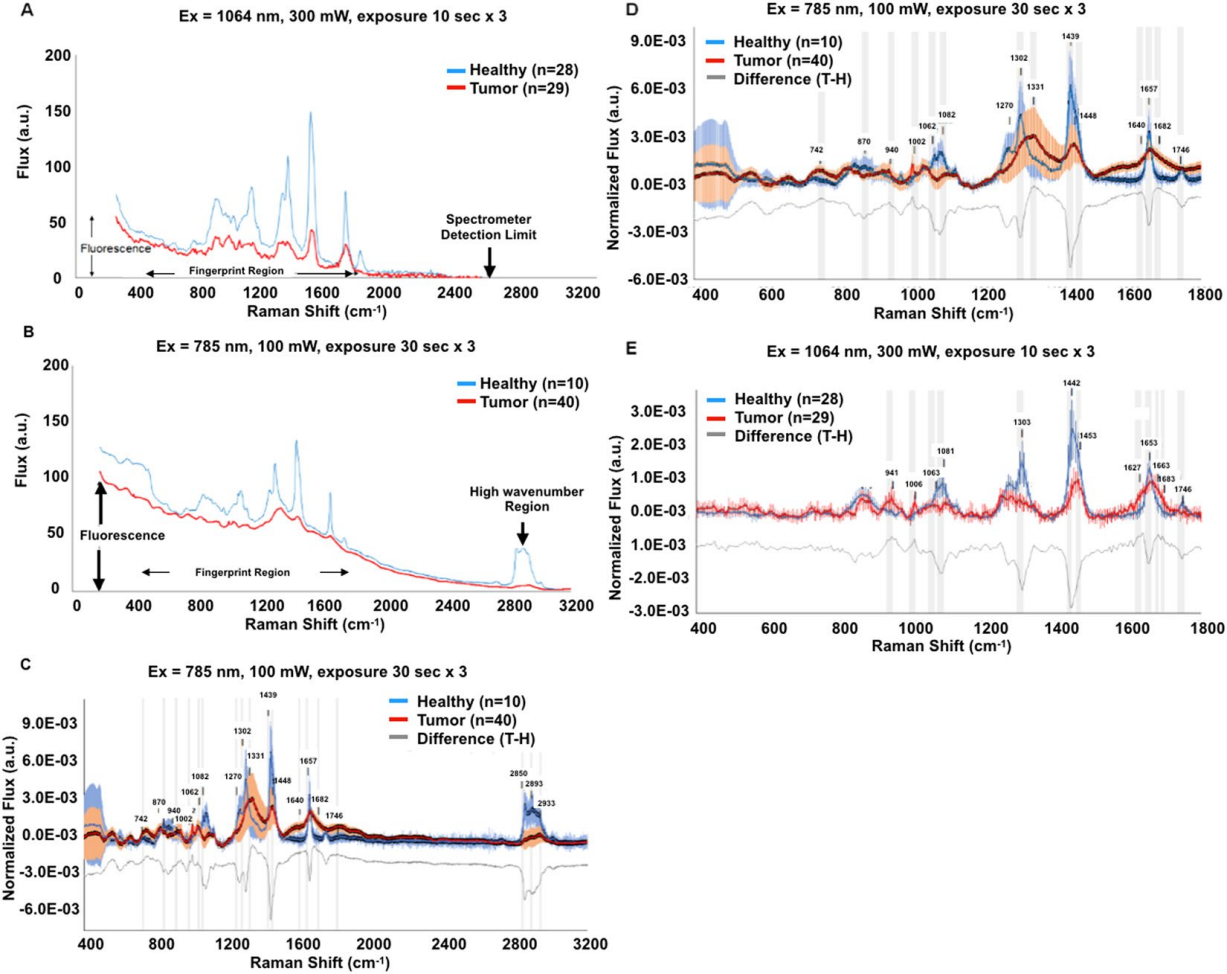
Raw Raman spectra can distinguish healthy and neoplastic tissue. Figure 2A and B compare the fluorescence generated by the two systems. The average raw Raman spectra for healthy and neoplastic tissue samples acquired using 1064 nm (**A**) and 785 nm (**B**) excitation wavelengths are presented exactly as collected without smoothing, fluorescence correction or area normalization. Total laser exposure (defined as laser excitation power x collection time) was 9 × 10^3^ mW-seconds for both systems. Raman scattering data are reported in counts per second. The 1064 nm system exhibits less than half the fluorescence (**A**) generated by the 785 nm device (**B**). Fluorescence-corrected, normalized Raman spectra of healthy and neoplastic tissue following 785 nm and 1064 nm excitation appear in (**C**,**D)** and in (**E**), respectively. Full Raman shift spectra provided by the 785 nm device appear in (**C**). The strong Raman signal generated in the high wavenumber region by healthy tissue decreases significantly in the signals generated by malignant tissue. Comparison of tumor and healthy signals reveals a malignant spectral signature in normalized Raman spectra. Raman bands contributing to the signatures are marked graphically by gray bands and listed in Table 1 for both systems. (**C**,**D**) and (**E**) also exhibit a difference spectrum (gray line), highlighting the disparities between the average healthy and cancerous signatures. Positive deviations from neutral mark increased flux in tumor spectra, while negative deviations denote increased flux in healthy spectra. Due to the limited detector size of the 1064 nm system, the Raman spectrum high wavenumber region (2800–3200 cm^−1^) can only be acquired using the 785 nm device.

Our first evaluation focused on the impact of tissue fluorescence on Raman signatures. Two tissue samples resected during breast conserving surgery for breast cancer were analyzed, one using the 785 nm system, the other using the 1064 nm device. All tumor spectra were collected from specimens containing invasive ductal carcinoma of the breast. Figure 2A depicts the average of the raw spectral data generated by the 1064 nm system for healthy (n = 28) and cancerous (n = 29) sites. Figure 2B depicts the average of the raw spectral data collected by the 785 nm system for healthy (n = 10) and tumor (n = 40) targets. Both systems exhibit a broad fluorescence offset that becomes increasingly pronounced below 400 cm^−1^, but the interference is more pronounced in the 785 nm system. At the start of the Raman fingerprint region (~400 nm), the 1064 nm system produces a fluorescence background level of ~39.9 counts per second (cps) compared to 83.7 cps for the 785 nm system.

Following fluorescence correction and area normalization, spectra for the two systems were evaluated for the presence of features distinguishing malignant from healthy tissue. Two regions in the Raman spectra are of potential interest for cancer diagnostics: the Raman fingerprint region (FP, 400–1800 cm^−1^) and the high wavenumber region (HW, 2800–3200 cm^−1^). The FP region provides information on the complex interactions between multiple bonds including a strong peak at 785 cm^−1^ associated with DNA and RNA nucleotides, broad peaks around 840 cm^−1^ and 941 cm^−1^associated with both collagen and glycogen, a sharp peak at 1004 cm^−1^ associated with the aromatic amino acid phenylalanine, another strong peak at 1092 cm^−1^ associated with the PO_4_ backbone, and bands characterizing lipid concentration and protein secondary structure such as the Amide I (C=O stretch near 1650 cm^−1^), Amide II (N–H bend + C–N stretch near 1550 cm^−1^) and Amide III bands (C–N stretch + N–H bend near 1300 cm^−1^), see Table 1 for a listing of bands of interest in breast cancer detection. The HW signal originates in the symmetric and asymmetric stretching vibrations of C-H bonds found in lipids, glycogen, proteins, RNA, and DNA (listed in order of Raman shift left to right in HW; see Mourant *et al*. 2005). Tissue composition studies using Raman spectroscopy have been reported, and molecular signatures have been identified for major cellular constituents. Lipid peaks indicating the presence of fat appear at 1267, 1301, 1444, and 1450 cm^−1^ ^18,30,31^. Carbon-hydrogen stretching in lipids result in broad peaks between 2800 cm^−1^ and 3000 cm^−1^, specifically at 2854, 2888, 2926, 2940, and 3009 cm^−1^ ^18,30,32^. Proteins also contribute to peaks in this region, specifically at 2905 cm^−1^ for non-acetylated C-H vibrations and at 2942 cm^−1^ for acetylated functional groups in malignant cells^8^. Amino acids from proteins exhibit peaks at 1243, 1245, 1265, 1305, 1430, 1653, 1663, and 1671 cm^−1^^30,33–35^. Carotenoids, levels of which are altered in malignant tissue relative to healthy tissue, have pronounced peaks at 1004, 1006, 1152, 1158, 1259, and 1518 cm^−1^ ^22,30,34,36–41^. Degree of vascularization associated with tumor growth may be measurable via the hemoglobin peak at 1560 cm^−1^^38^.

**Table 1:** Most likely Raman band assignments of interest in this study for breast cancer diagnostics^65,69–78^.

Raman Shift (cm^−1^)	Origin
742–749	Ring breathing mode of DNA and RNA bases, symmetric breathing of tryptophan (protein assignment)
858–882	C-C stretching mode from multiple sites in collagen backbone, α-helix, valine, proline
938–950	Proline, *ν*(C-C) skeletal of collagen backbone, polysaccharides including C-O-C skeletal mode
1002–1004	*ν*_*s*_(C-C) phenylalanine ring breathing mode
1062–1063	Chain C-C stretch in lipids; C-O and C-N stretch in proteins; O-P-O stretch in DNA and RNA
1081–1082	Nucleic acids; C-C and C-O stretching modes in phospholipids
1271–1278	Amide III (α-helix), collagen
1302–1303	lipids *δ*(CH_2_) twisting of lipids, fatty acids, and/or collagen
1325–1333	DNA, phospholipids
1439–1442	CH_2_ bending mode in normal breast tissue
1448–1453	CH_2_ bending mode in malignant breast tissue
1627–1640	Amide I
1653–1657	C=C of lipids in healthy tissue, not the amide I
1662–1667	Nucleic acid modes; indicator of tissue DNA content, amide I
1683–1697	Amide I disorder structure, collagen
1745–1750	*ν* (C=O) stretch in phospholipids; C=O stretch of lipids in normal tissue
2850–2875	CH_2_ symmetric stretch of lipids; CH_2_ asymmetric stretch of lipids + proteins
2885–2908	CH_2_ asymmetric stretch of lipids and proteins
2945–2957	CH_3_ asymmetric stretch of proteins; aliphatic and aromatic CH stretching vibrations in nucleic acids

Note: Peak assignments are given as a range covering the mean values generated by the two Raman devices.

HW only appears using the 785 nm system. The 1064 nm system’s limited spectral bandwidth prohibits collection of HW data. Figure 2C–E depict the average fluorescence corrected and area normalized spectra with 95% confidence intervals for the two devices [for Ex = 1064 nm, healthy (n = 28), tumor (n = 29); for Ex = 785 nm healthy (n = 10), tumor (n = 40)]. The figure includes the difference between the two averages (Difference = Tumor – Healthy) for both systems. Figure 2C presents the full 400–3200 cm^−1^ spectra for the 785 nm system including the HW region (2800–3200 cm^−1^). Gray vertical bands highlight 17 regions for the 785 nm spectra (Fig. 2D) and 12 regions on the 1064 nm spectra (Fig. 2E) that show significant (1-sigma) differences distinguishing healthy from cancerous spectra, and are free from contamination by surgical dyes.

Some peaks appear unique for each Raman device. The 785 nm data shows a pronounced increase in strength of the 742 cm^−1^ band and the appearance of a strong band at 1330 cm^−1^ for neoplastic tissue (Fig. 2D). The 1064 nm system identifies a broad band at 941 cm^−1^ that is significantly enhanced in malignant tissue compared to the levels found in healthy sites (Fig. 2E). Following analysis of the 95% confidence interval overlap between the spectra and with qualitative consideration of the difference spectrum for the two target classes (benign and malignant), the 12 bands in the 1064 nm data and 17 bands in the 785 nm data were used to test the classification performance of the two systems. (To review the biomolecular assignments for Raman activity in these regions see Table 1).

### Multivariate exploratory analysis for regions of interest using principal component analysis

PCA loadings generated by multivariate analysis of the correlations for the 12 bands from the 1064 nm system and 17 bands from the 785 nm device appear in Tables 2 and 3. Data were acquired from three experimental configurations: the 1064 nm and 785 nm systems each using a microscope for laser excitation and collection of scattered light, and then the 785 nm system using only the hand-held probe appropriate for use in a surgical setting.

**Table 2:** Principal component analysis (PCA) extracts 6 eigenvectors accounting for >99% of the variance in 12 bands from the 1064 nm system and 17 bands from the785 nm device. Eigenvector loadings >0.4 (+ or −) for each PC appear in bold.

1064 nm (Microscope)	PC1	PC2	PC3	PC4	PC5	PC6
**Eigenvalue**	2.31E-06	1.33E-07	2.71E-08	1.14E-08	8.67E-09	6.75E-09
**Percent**	91.8	5.2	1.1	0.5	0.3	0.3
**Cum Percent**	91.8	97.0	98.1	98.6	98.9	99.2
**Bands**	**Loadings**					
B_941	−0.117	0.261	**0.600**	−0.060	**0.407**	**−0.549**
B_1006	−0.094	0.294	0.144	**0.639**	−0.301	−0.067
B_1063	0.146	−0.053	**0.503**	0.134	0.104	**0.561**
B_1081	0.246	−0.118	0.328	−0.036	−0.316	0.148
B_1303	**0.440**	−0.123	0.126	−0.025	0.238	−0.036
B_1442	**0.633**	0.020	0.029	−0.200	−0.081	−0.302
B_1453	**0.468**	0.236	−0.145	**0.509**	0.158	0.067
B_1627	−0.135	0.190	0.017	0.045	**0.498**	0.369
B_1653	0.168	0.363	−0.331	−0.271	0.322	0.198
B_1663	0.087	**0.640**	−0.074	−0.172	−0.355	0.017
B_1683	−0.137	**0.415**	0.238	−0.275	−0.143	0.156
B_1746	0.096	−0.088	0.222	−0.296	−0.219	0.245
**785 nm (Microscope)**	**PC1**	**PC2**	**PC3**	**PC4**	**PC5**	**PC6**
**Eigenvalue**	1.01E-05	7.94E-07	1.64E-07	5.94E-08	2.95E-08	1.79E-08
**Percent**	90.2	7.0	1.5	0.5	0.3	0.2
**Cum Percent**	90.2	97.2	98.7	99.2	99.5	99.7
**Bands**	**Loadings**					
B_742	−0.074	−0.058	−0.152	0.196	0.167	−0.179
B_870	0.104	−0.023	−0.040	0.303	−0.364	0.253
B_941	−0.015	−0.162	**0.466**	0.222	**−0.448**	0.083
B_1002	−0.091	−0.244	0.324	0.265	**0.643**	0.031
B_1062	0.209	−0.206	0.041	0.172	0.122	**0.541**
B_1082	0.233	−0.157	0.013	0.263	0.105	0.265
B_1302	0.309	**0.522**	−0.129	**0.577**	0.020	−0.031
B_1331	−0.137	**0.739**	0.196	−0.089	0.196	0.196
B_1439	**0.575**	0.012	−0.099	**−0.467**	0.203	0.225
B_1448	0.390	0.102	**0.548**	−0.224	−0.187	−0.043
B_1640	0.109	0.047	0.285	0.042	0.022	−0.178
B_1657	0.178	0.002	0.250	0.102	0.020	**−0.402**
B_1682	−0.167	0.013	0.236	0.001	0.157	0.168
B_1746	−0.007	0.022	−0.119	0.088	−0.024	0.141
B_2850	0.322	−0.071	−0.215	0.098	−0.037	−0.310
B_2893	0.269	−0.052	−0.028	0.099	0.093	−0.251
B_2933	0.183	−0.055	0.161	0.045	0.222	−0.204

**Table 3:** Principal component analysis (PCA) extracts 6 eigenvectors accounting for >99% of the variance in bands from the785 nm device. Data were collected using only the hand-held probe instead of a microscope. Total laser exposure time for each target was 10 seconds. Eigenvector loadings >0.4 (+ or −) for each PC appear in bold.

785 nm (Handheld Probe)	PC1	PC2	PC3	PC4	PC5	PC6
**Eigenvalue**	1.2E-05	2.14E-07	1.78E-07	9.37E-08	4.81E-08	4.02E-08
**Percent**	95.0	1.7	1.4	0.7	0.4	0.3
**Cum Percent**	95.0	96.7	98.1	98.8	99.2	99.5
**Bands**	**Loadings**					
B_742	−0.075	**0.558**	−0.126	**0.755**	−0.024	−0.136
B_870	0.043	−0.174	−0.106	0.021	0.314	0.293
B_941	−0.062	−0.345	0.018	0.217	0.100	0.143
B_1002	−0.065	−0.342	−0.035	0.357	−0.002	0.063
B_1062	0.076	−0.250	−0.282	0.094	0.391	−0.212
B_1082	0.136	−0.087	−0.323	0.182	**0.415**	0.116
B_1302	0.332	**0.469**	0.055	−0.168	0.224	0.350
B_1331	0.048	0.101	**0.683**	0.099	**0.472**	−0.175
B_1439	**0.581**	−0.122	0.234	0.003	−0.008	−0.178
B_1448	**0.433**	−0.223	0.164	0.240	−0.167	−0.207
B_1640	−0.028	−0.004	0.184	0.184	−0.258	0.293
B_1657	0.271	−0.067	0.050	0.094	−0.261	**0.518**
B_1682	−0.066	−0.182	0.207	0.226	−0.189	0.144
B_1746	0.034	−0.019	0.032	0.132	0.244	0.371
B_2850	0.339	0.105	−0.250	−0.028	−0.047	−0.066
B_2893	0.301	0.079	−0.263	−0.026	−0.083	0.043
B_2933	0.195	−0.048	−0.156	0.066	−0.161	−0.257

Six eigenvectors (PC1-PC6) accounting for >99% of the variance in 12 bands from the 1064 nm system and 17 bands from the 785 nm device were extracted by Principal Component Analysis (PCA). Eigenvector loadings >±0.4 have been highlighted in bold to give a qualitative indication of important contributors to the discrimination of these spectra. The first 3 PCs account for >98.0% of the variance in both the 1064 nm and 785 nm data.

For the 1064 nm data, PC1 includes strong contributions from bands at 1443 cm^−1^ and 1453 cm^−1^, spectral regions assigned to CH_2_ bending modes in normal and malignant tissue, and the 1303 cm^−1^ band assigned to *δ*(CH_2_) twisting of lipids, fatty acids, and/or collagen. PC2 includes information from 1663 cm^−1^ assigned to nucleic acid modes, and 1683 cm^−1^ assigned to amide I disorder and collagen. PC3 contains information from 941 cm^−1^ assigned to collagen backbone and polysaccharides, and 1063 cm^−1^ assigned to O-P-O stretch in DNA and RNA. PC4 includes contributions from 1006 cm^−1^ assigned to *ν*_*s*_(C-C) phenylalanine ring breathing mode and 1453 cm^−1^ assigned to CH_2_ bending modes in malignant tissue. PC5 represents information from 1627 cm^−1^ assigned to amide I, and 941 cm^−1^ assigned to collagen backbone and polysaccharides. PC6 is dominated by contributions from 1063 cm^−1^ assigned to O-P-O stretch in DNA and RNA and 941 cm^−1^ assigned to collagen backbone and polysaccharides.

For the 785 nm microscope data, PC1 includes strong contributions from 1439 cm^−1^, a spectral region assigned to CH_2_ bending modes in normal breast tissue. PC2 includes information from 1331 cm^−1^ assigned to DNA and phospholipids, and 1302 cm^−1^ assigned to *δ*(CH_2_) twisting of lipids, fatty acids, and/or collagen. PC3 contains information from 1448 cm^−1^ assigned to CH_2_ bending modes in malignant breast tissue and 941 cm^−1^ assigned to collagen backbone and polysaccharides. PC4 includes contributions from 1302 cm^−1^ assigned to *δ*(CH_2_) twisting of lipids, fatty acids, and/or collagen and 1439 cm^−1^ assigned to CH_2_ bending modes in normal breast tissue. PC5 represents information from 941 cm^−1^ assigned to collagen backbone and polysaccharides. PC6 is dominated by contributions from 1657 cm^−1^ assigned to the C=C of lipids in healthy tissue.

For the 785 nm handheld probe data (Table 3), PC1 includes strong contributions from the 1439 cm^−1^ and 1448 cm^−1^ bands, spectral regions assigned to CH_2_ bending modes in normal and malignant breast tissue, respectively. PC2 includes information from 742 cm^−1^ a region that can be assigned to the ring breathing mode of DNA and RNA bases, or the symmetric breathing of tryptophan, and 1302 cm^−1^ assigned to *δ*(CH_2_) twisting of lipids, fatty acids, and/or collagen. PC3 contains information from 1331 cm^−1^ assigned to DNA and phospholipids. PC4 includes a strong contribution from 742 cm^−1^ assigned to the ring breathing mode of DNA and RNA bases and/or the symmetric breathing of tryptophan. PC5 represents information from 1331 cm^−1^ assigned to DNA and phospholipids and 1082 cm^−1^ assigned to nucleic acids as well as the C-C and C-O stretching modes in phospholipids. PC6 is dominated by contributions from 1657 cm^−1^ assigned to the C=C of lipids in healthy tissue.

### Raman classification of putative healthy and neoplastic breast tissue by linear discriminant analysis

The first 3 PCA factors accounting for more than 98% of the variance in the data for both the 1064 nm and 785 nm systems were used as inputs for Linear Discriminant Analysis (LDA) classification. The combination of these multivariate techniques for feature extraction and classification will be referred to as PCA-LDA. Bands employed to generate the principal components used by LDA refer to those displayed in Table 1.

**Figure 3:**
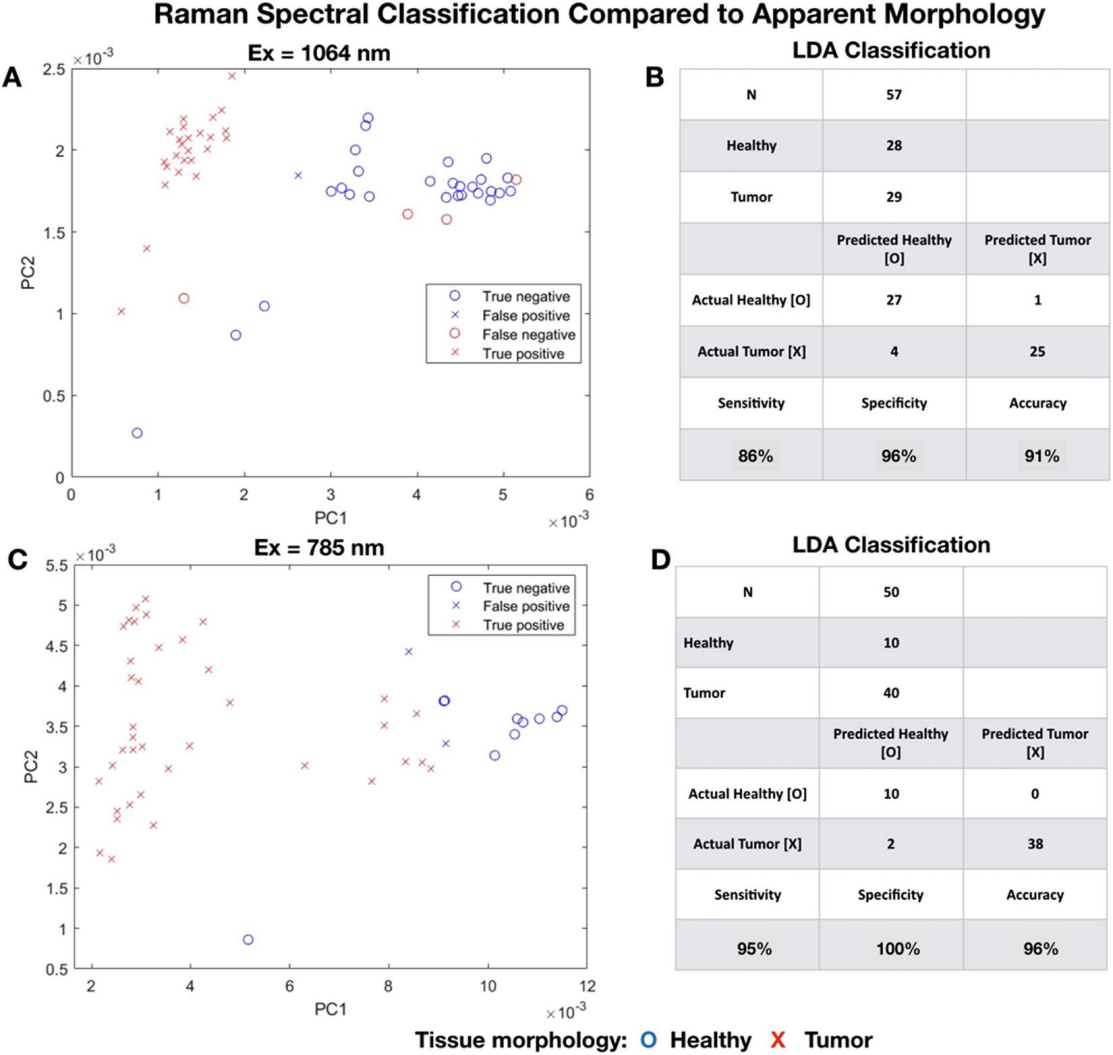
PCA-LDA classification of Raman spectra generated by 1064 nm and 785 nm systems. Figure 3 depicts the PCA-LDA identification of two spectral classes for tissue regions that by visual morphological classification were either tumor-rich () or healthy (). We utilized the 3 PCA factors (Table 3) extracted from the 1064 nm and 785 nm data as inputs for LDA classification. Figure 3A is a plot of PC1 and PC2 factors extracted from 1064 nm spectral data from 57 targets in tissue regions that appeared either macroscopically healthy (N = 28) or tumor-rich (N = 29). LDA (Fig. 3B) classifies 27 of the 28 spectra from healthy regions as healthy, and 25 of 29 spectra from tumor-rich regions as pathological (sensitivity = 86%, specificity = 96%, and accuracy = 91%). Figure 3C is a plot of PC1 and PC2 factors extracted from 785 nm data from 50 targets in tissue regions that appeared either macroscopically healthy (N = 10) or tumor-rich (N = 40). LDA (Fig. 3D) classifies 10 of the 10 spectra from healthy regions as healthy, and 38 of 40 spectra from tumor-rich regions as pathological (sensitivity = 95%, specificity = 100%, and accuracy = 96%).

Figure 3 depicts the PCA-LDA identification of two spectral classes for tissue regions that by visual morphological classification were either tumor-rich () or healthy (). We utilized 3 PCA factors (Table 2) extracted from the 1064 nm and 785 nm data as inputs for LDA classification. Figure 3A is a plot of PC1 and PC2 factors extracted from 1064 nm spectral data from 57 targets in tissue regions that appeared either macroscopically healthy (N = 28) or tumor-rich (N = 29). LDA (Fig. 3B) classifies 27 of the 28 spectra from healthy regions as healthy, and 25 of 29 spectra from tumor-rich regions as pathologic (sensitivity = 86%, specificity = 96%, and accuracy = 91%). Figure 3C is a plot of PC1 and PC2 factors extracted from 785 nm data from 50 targets in tissue regions that appeared either macroscopically healthy (N = 10) or tumor-rich (N = 40). LDA (Fig. 3D) classifies 10 of the 10 spectra from healthy regions as healthy, and 38 of 40 spectra from tumor-rich regions as pathological (sensitivity = 95%, specificity = 100%, and accuracy = 96%).

**Figure 4:**
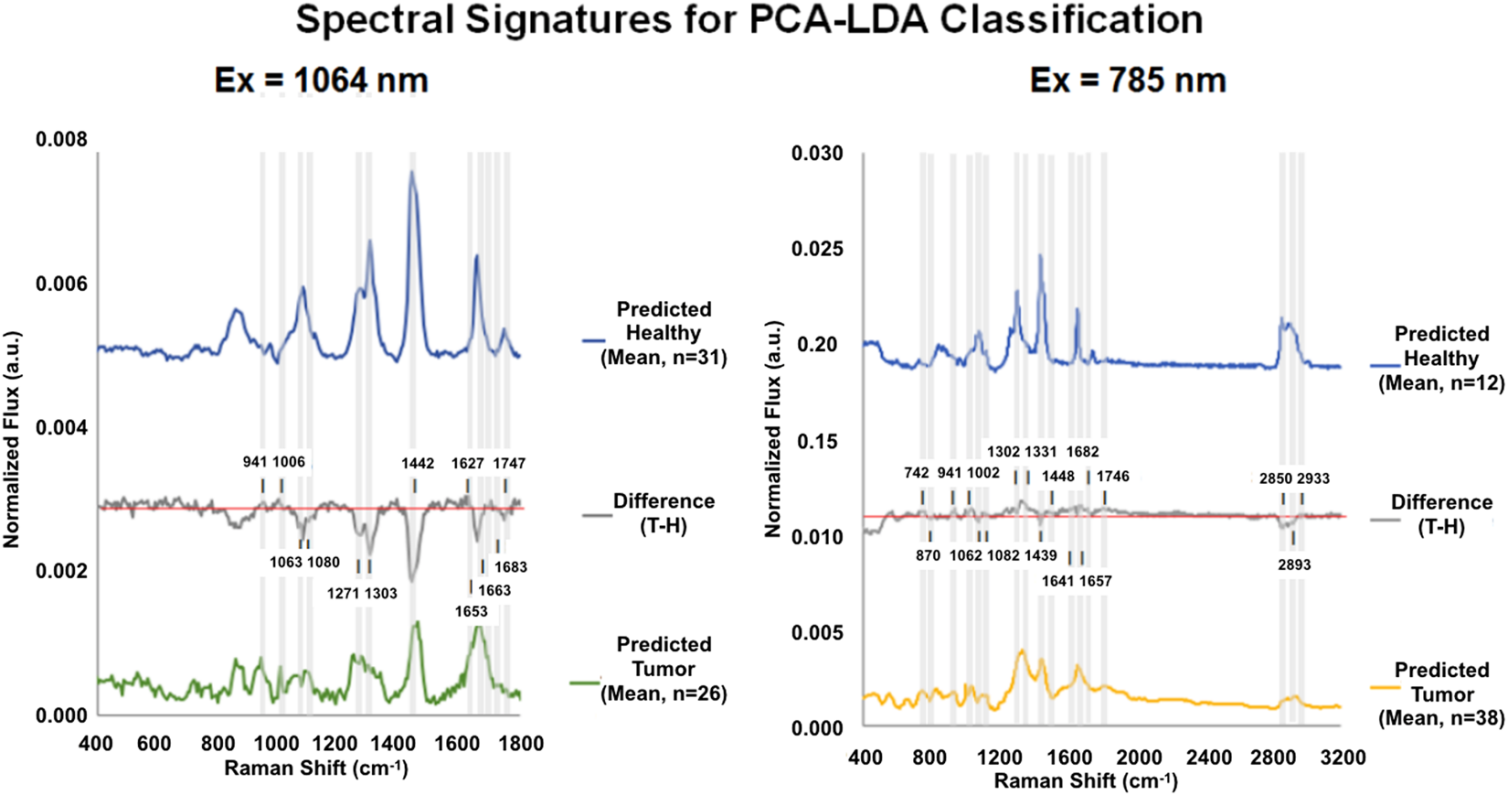
Average spectra for tissue classified as Healthy, Mixed, and Tumor by PCA-LDA. Spectra in (**A**) were generated using 1064 nm excitation. The figure depicts the average spectra for targets identified as Healthy and Tumor by PCA-LDA as well as the difference spectrum created by subtracting the Tumor (T) from the Healthy average spectra. The difference spectrum reveals increased flux for nucleic acid and protein bands at 941 and 1006 cm^−1^ as well as loss of signal strength at 1271, 1063, 1080, 1303, 1442, and 1653 cm^−1^ characteristic of shifts in protein and lipid species common in spectra from tumor-rich tissue. Spectra in (**B**) were acquired with the 785 nm system. Here the features in the difference spectrum distinguishing the Tumor from Healthy signatures are less pronounced than in the 1064 nm data, but once again there are increases in flux at 742, 941, and 1002 cm^−1^ attributed to increased nucleotide and protein tissue concentrations as well as a marked loss of flux at 2850 attributed to a decrease in lipid content.

Figure 4 depicts the average spectra for the targets in each of the classes identified by PCA-LDA. Figure 4 also displays two “difference spectra”, representing the difference between the tumor spectra found in healthy tissue and the average healthy spectrum. Values above the center axis indicate that tumor signal intensity for that particular spectral region is greater than the signal intensity of the healthy tissue. Values below the axis imply the healthy tissue Raman activity is greater than that of tumor cells.

### Margin characterization: Obtaining transit images and spectra while crossing from apparently healthy to tumor-rich tissue

When resecting tumors, the surgeon strives for achieving “negative margins”, i.e., complete excision of all malignant tissue such that no tumor cells are extending to the inked margins as assessed by microscopic pathologic evaluation. During that excision, healthy tissue surrounding the tumor is also removed. Determination of where cancer ends and healthy tissue begins is traditionally done by visual inspection of the tissue during surgery; however, margins or transitional regions may contain cancerous cells that have migrated out of the primary tumor in a fashion that is not visually detectable macroscopically; this could lead to unintentional residual tumor cells being left behind, which in turn cause cancer relapse. Thus, we inquired if Raman spectra could identify transitional tissue that may visually appear healthy but already be malignant in nature.

**Figure 5:**
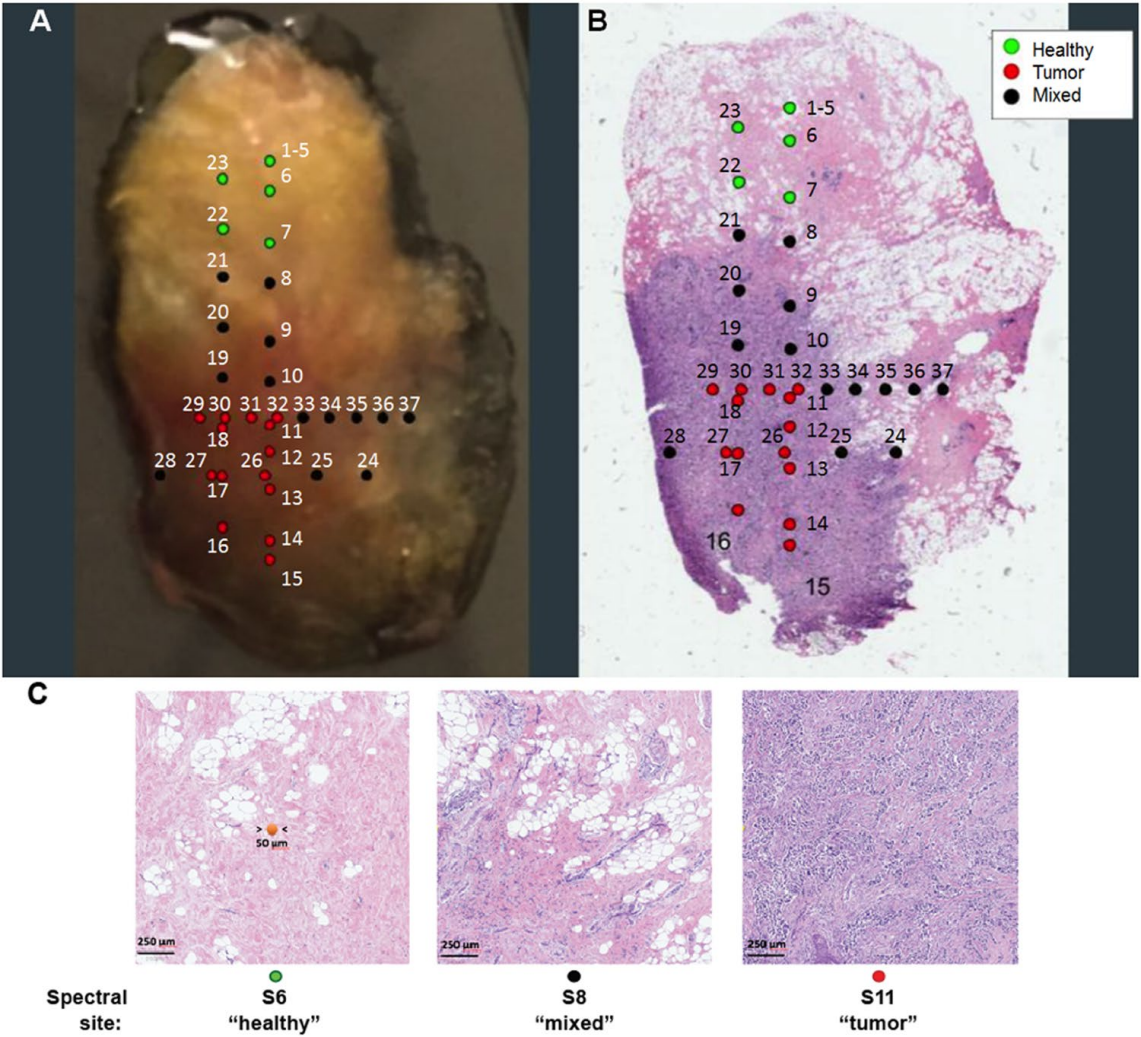
Representative tumor specimen and sites for collection of Raman spectra. The 785 system in microscope mode collected spectra along four transects designed to move sequentially across the visible boundaries between healthy and cancerous tissue. (**A**) Spectral collection sites along four transits pictured using a visible light image of an intact surgical specimen. Transit 1 sites are labelled 1–15; Transit 2 ran from 16 to 23; Transit 3 from 24 to 28; and Transit 4 from 29 to 37. Following data collection, the samples are fixed in formalin, embedded in paraffin, sectioned at 4 μm, and placed on glass slides for H&E staining. Whole slide images are obtained by scanning at 20X magnification. (**B**) The corresponding H&E stained section used for standard margin analysis is shown. Target sites are color-coded according to histological classification: green for healthy, red for regions dominated by tumor, and black for a mixture of healthy and cancerous tissue (“mixed”). (**C**) H&E photomicrographs for target sites s6 (green, healthy), s8 (black, mixed), and s11 (red, tumor) acquired during first transit depicted in (**A**) and (**B**). The Raman probe samples a circular area with a diameter of approximately 50–85 μm (in orange, a central spot representing the relative size of the laser beam). Refer to Fig. S1A for matching spectra.

In this experiment, the 785 nm system in microscope mode collected spectra along four transects designed to move sequentially across the visible boundaries between healthy and cancerous tissue. Figure 5A shows the transects and collection sites as they were acquired from the intact specimen Fig. 5B shows the transects and collection sites against the H&E stained specimen. The pathologist in our group (DS) evaluates a 1 mm^2^ area of tissue on the H&E image surrounding each putative target site and scores the region as healthy, tumor, or mixed, with the latter classification meaning that the area clearly contains a mixture of both healthy and tumor cells. Target locations and 1 mm^2^ surrounding regions were annotated and regions of interest (ROI) were mapped on the photomicrograph of the H&E image using QuPath.

Figure 5C shows the H&E photomicrographs of the 1 mm^2^ region around targets s6 (Fig. 5C, *left*, healthy), s8 (Fig. 5C, *middle*, mixed), and s11 (Fig. 5C, *right*, tumor). The Raman probe samples a circular area with a diameter of approximately 50–85 μm. Figure 5C displays a central spot representing the relative size of the laser beam.

**Figure 6:**
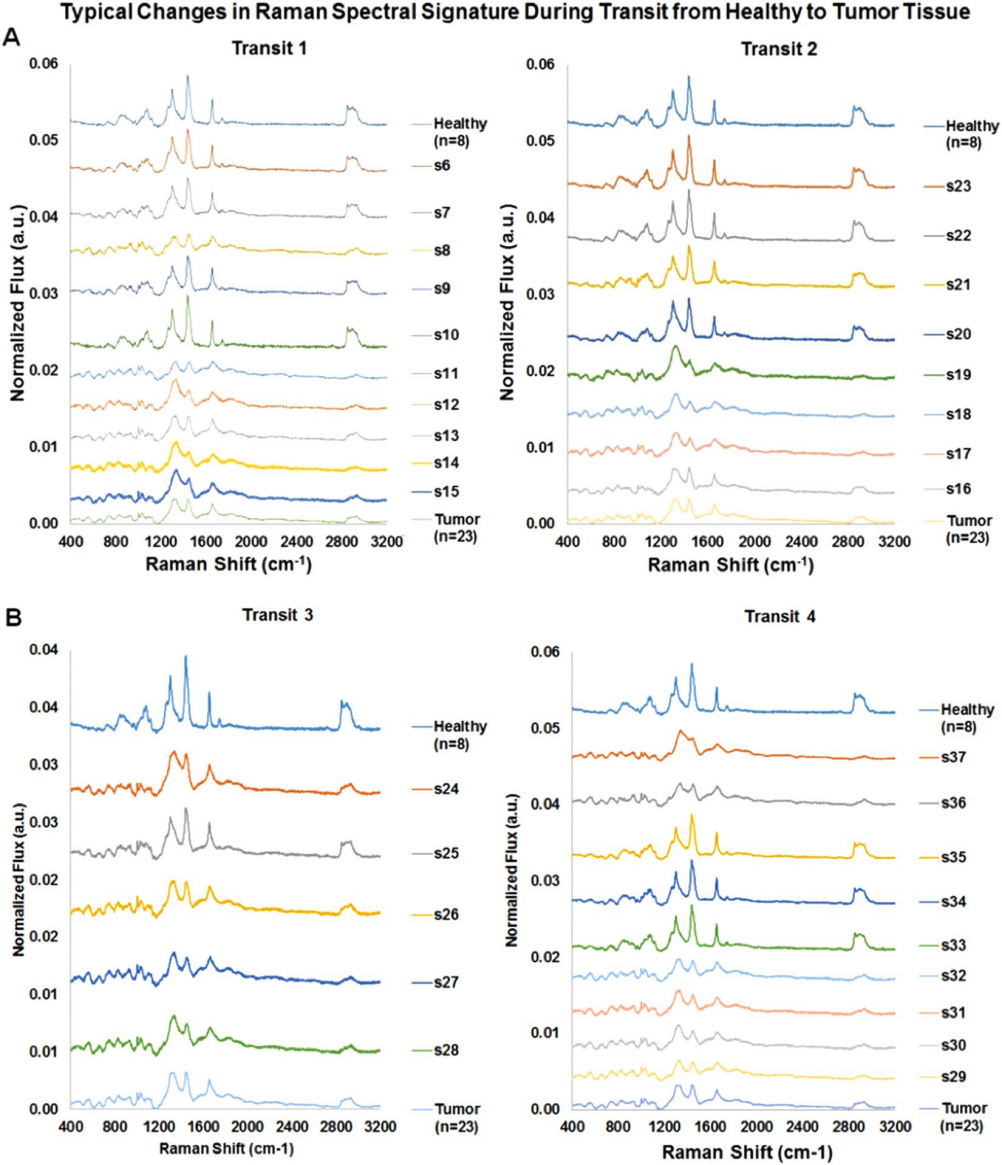
Typical Changes in Raman Spectral Signatures during multiple data collection transits from healthy tissue to tumor tissue. A series of Raman spectra were obtained at ~1 mm intervals along a straight line moving from healthy to tumor tissue (or vice-versa). Such a series was termed a “transit”. By definition, each transit crosses the boundary between the two regions. Raman spectra for tissue sites along four transits are depicted. Each spectrum is the average of three scans, each with an integration time of 30 seconds. Total laser exposure time for each sample is 90 seconds. XY-coordinates for target location are recorded using the microscope micrometer. Spectra identifiers refer to target site designations depicted in Fig. 5. Spectra are numbered in temporal order of collection. For ease of viewing, spectra in Fig. 5 are offset and ordered (from top to bottom of the page) from data collected in putatively healthy tissue, across a boundary region, and then on into a tumor-rich region. For reference, the average spectra obtained from healthy (n = 88) and cancerous (n = 23) tissues, are depicted at the top and bottom, respectively, for each transit. Spectra s1-s5 were collected prior to first transit to evaluate signal/noise characteristics and are discussed in the supplemental material. Transit 1 starts with healthy spectra at sites s6 and s7. There is a clearly abnormal signature at s8, followed by a return to healthy spectra at sites s9 and s10. A clear shift to neoplastic spectra starts at s11 continuing through s15. Transit 2 starts with neoplastic signatures for sites s16-s19, then shifts to healthy spectra for sites s20-s23. Transit 3 traversed a region that appeared to be a mixture of tumor and healthy tissue in both the visible light and H&E images. All of the spectra (s24 through s28) appear to be a mixture of tumor and healthy signatures. Transit 4 starts in a tumor-rich region with spectra at s29-s32 closely resembling the average tumor spectra. The spectra then changes to a series of healthy tissue signatures at sites s33-s35, and finally shifts back to a tumor signature at sites s36 and s37.

Figure 6 depicts the Raman spectra obtained during each transit in Fig. 5. Spectral labels (s6 through s37) refer to the sites labeled in both the visible light (Fig. 5A) and H&E (Fig. 5B) images. In Transit 1 (Fig. 6A, *left panel*), spectra s1- s5 (not shown here; see supplement data) plus spectra from sites s6 and s7 were acquired in what appeared macroscopically in visible light to be pale yellow healthy tissue. Morphological data from H&E stains (Fig. 5B,C) and the Raman spectral data shown here support that clinical impression. The exact transition from healthy to cancer tissue for this transit is difficult to pinpoint in using only reflected visible light information (Fig. 5A). Fingers of red and orange arch up to intersect with the site of spectrum s8. A clear color shift occurs between targets s9 and s10. Histological examination revealed that s7, s8, and s9 contained mixtures of healthy and tumor cells (Fig. 5C depicts s8 histology). Figure 6A depicts healthy spectra at sites s6 and s7, an abnormal signature at s8, and a return to healthy spectra at sites s9 and s10. A shift to neoplastic spectra starts at s11 continuing through s15. Macroscopic visual examination, histology, and spectral data all classify sites s11–s15 as tumor-rich.

For Transit 2 the visible light image, H&E data, and Raman probe all agree that sites s16, s17, and s18 are tumor-rich and sites s22 and s23 are healthy. The reflected light image indicates transition from tumor to healthy tissue should occur somewhere between s20 and s21. H&E staining photomicrographs find a mixture of tissues at sites s19, s20, and s21. The Raman for site s19 appear more similar to the average tumor spectrum, while spectra for sites s20 and s21 are closely matched to the average healthy spectrum.

For Transit 3 (Fig. 6B, *left panel*) visual inspection revealed only one small area of potentially healthy tissue at s24. H&E stains identify both healthy and tumor cells in this region. The Raman spectrum shows changes in the fingerprint and high wavenumber regions characteristic of a mixture of tumor and healthy. For Transit 4 visual inspection, Raman spectra and histology code sites s29, s30, s31, s32 as tumor (Fig. 6B, *right panel*). For the five remaining sites (s33 to s37) visible light images shows a gradual shift from dark red-brown near the center of the sample to a light yellow and green at the periphery. The H&E stain shows a patchwork of red and purple indicating that the region is a mixture of healthy and tumor tissues. The Raman spectra show relatively strong lipid signatures from sites s33, s34, and s35, while spectra from sites s36 and s37 clearly exhibit spectral signatures characteristic of tumor.

The data generated during these 4 transits suggest a strong correlation between Raman spectral signatures and histological imaging when Raman data are acquired with the aid of a laboratory microscope and the data are collected for 90 seconds. Since the target application for this technology is handheld tumor margin examination during surgical intervention, we next explored the ability of the system to discriminate malignant from healthy tissue using only the system probe head (no microscope) and with data collection time limited to 10 seconds.

### Tissue classification using raman spectra collected without microscope

The i-Raman probe head when removed from the microscope can either be used hand-held in the operating theater employing an embedded trigger to initiate spectral acquisition, or it can be securely fastened into a small stand (part BAC150B, probe holder) with an integrated XY-stage to systematically interrogate excised samples while documenting XY-coordinates. For this experiment, data acquisition was accomplished with a single 10 second scan using the bare probe secured in the probe holder.

**Figure 7:**
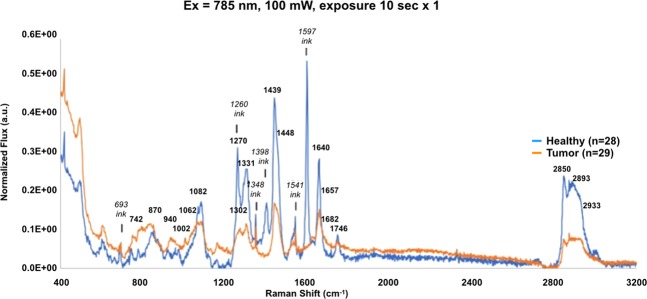
Rapid characterization of tumor and healthy tissue using only the i-Ramani-Raman probe head without microscope. Spectral data were acquired with a single 10 second scan using the bare i-Ramani-Raman probe (785 nm excitation), uncoupled from the microscope, and secured in the probe holder. Spectral data shown are the averages of the 28 healthy and 29 tumor region spectra, where the fluorescence was corrected and the resulting spectra area normalized. Raman bands detecting significant activity from surgical marking ink are noted (693, 1260, 1348, 1398, 1541, and1597 cm^−1^).

Figure 7 shows the average of 28 healthy and 29 tumor region spectra. This was the first tissue sample exhibiting a significant Raman signature for the surgical marking ink commonly used to provide landmarks for pathology. Prominent Raman-active modes for the ink can be seen at 693, 1260, 1348, 1398, 1541, and 1597 cm^−1^. Of the 17 spectral regions of interest in the 785 nm system for detecting cancer, the ink currently in use in our operating theater only compromises data collection for the 1260 cm^−1^ band. Data analysis is accomplished using 3 bands from the high wavenumber region and 13 bands from the fingerprint region, omitting all data from the contaminated cm^−1^ band.

**Figure 8:**
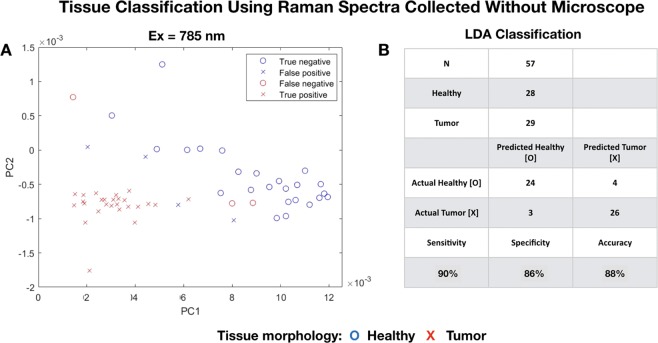
Tissue Classification Using Raman Spectra Collected Without Microscope. Figure 8 depicts the PCA-LDA identification of two spectral classes for tissue regions that by visual morphological classification were either tumor-rich () or healthy (). We utilized 3 PCA factors (Table 3) extracted from the 785 nm data as inputs for LDA classification. Fig. 8A is a plot of PC1 and PC2 factors extracted data from 57 targets in tissue regions that appeared either macroscopically healthy (N = 28) or tumor-rich (N = 29). LDA (**B**) classifies 24 of the 28 spectra from healthy regions as healthy, and 26 of 29 spectra from tumor-rich regions as pathological (sensitivity = 90%, specificity = 86%, and accuracy = 88%).

Figure 8 depicts the PCA-LDA identification of two spectral classes for tissue regions that by visual morphological classification were either tumor-rich () or healthy (). We utilized 3 PCA factors (Table 3) extracted from the 785 nm data as inputs for LDA classification. Figure 8A is a plot of PC1 and PC2 factors extracted data from 57 targets in tissue regions that appeared either macroscopically healthy (N = 28) or tumor-rich (N = 29). LDA (Fig. 8B) classifies 24 of the 28 spectra from healthy regions as healthy, and 26 of 29 spectra from tumor-rich regions as pathological (sensitivity = 90%, specificity = 86%, and accuracy = 88%).

## Discussion

There are two core observations in this set of experiments. First, off-the-shelf laser Raman probes sufficiently compact for use in a spatially limited surgical field can acquire Raman diagnostic data distinguishing cancerous from healthy breast tissue in 10–90 seconds. Second, the PCA-LDA analysis employed here made relatively minimal use of the HW information. While the dramatic loss if signal in the HW region of the Raman spectra may be able to serve as a preliminary predictor of the full spectrum diagnostic effort, there is one clear caveat. Although the HW region contains lipid, glycogen, protein, and RNA/DNA information, it is a region primarily characterized by a loss of signal strength as the probe moves from healthy to tumor tissue. It is not a region where a relatively weak signal from healthy tissue transforms into a strong signal from the massive increase in peri-nuclear proteins, DNA, and RNA characteristic of neoplastic breast tissue. We suggest that the HW region may serve as a useful warning signal of tissue damage and certainly deserves further investigation, the focus needs to remain on signal deconvolution of multiplexed nucleotide and protein signatures in both the fingerprint and HW regions^4,42–46^.

In comparing these commercial instruments, both are portable, easy to use, and required no special modifications for use as a diagnostic. 1064 nm systems have been previously shown to be successful in cancer diagnostics^34,37,38,47^ and produce less fluorescence than 785 nm devices in select biological targets. Efforts to minimize fluorescence masking of Raman signatures occupy a significant amount of investigator time and have spawned an array of suppression techniques^48–54^. We agree that minimizing the original fluorescence signal from the target is the preferred route rather than relying on post-acquisition data processing. Our experiments show that the longer wavelength, lower energy 1064 nm system certainly generates less fluorescence activity in healthy and malignant breast tissue than does the 785 nm device. However, the 785 nm spectrometer exhibits significant advantages in spectral range and resolution. The spectra produced by the 1064 nm system spans approximately 2200 cm^−1^ with a spectral resolution of 5.3 cm^−1^. The 785 nm device covers just over 3000 cm^−1^ with a resolution of 1.7 cm^−1^ per sample (See Table 4).

**Table 4:** Operating characteristics for the i-Raman Plus and i-Raman EX systems.

Device	Wavelength (nm)	Detector (Pixels)	Effective Pixels	Range (cm^−1^)	Bandwidth (cm^−1^)	Resolution (cm^−1^)
i-Raman Plus	785	2048	1804	175–3201	~3026	1.78
i-Raman EX	1064	512	428	247–2500	~2253	5.07

While an ideal instrument for minimizing fluorescence and maximizing Raman information content may ultimately turn out to be a 1064 nm spectrometer with a 200–3200 cm^−1^ bandwidth, the fundamental physics of photonic detectors poses a significant engineering and financial difficulty. To acquire Raman shift data between 200 and 3200 cm^−1^, the detector for a 1064 nm spectrometer must efficiently collect photons ranging in wavelength space from ~1087 nm to ~1613 nm. For a 785 nm system, the lower and upper detection bounds in real wavelength space are only ~797 nm and ~1048 nm. The efficiency of silicon-based detectors falls off rapidly after ~1000 nm. As a result, while detectors for a 785 nm device can use relatively inexpensive silicon-based components, detectors required to operate from 1000–1800 nm for the 1064 nm systems must use much more expensive InGaAs (Indium gallium arsenide) chips capable of more efficient performance beyond 1000 nm. Wide-spread availability of affordable 1064 nm Raman spectrometers with full spectrum bandwidth must await improvement in cost-effective manufacturing techniques for larger InGaAs sensors.

The 50–85 μm beam size of most commercial Raman spectrometers including the ones tested here is an excellent match for clean detection of approximately 15–20 clustered tumor cells, approximately the same number of cells required for reliable histological diagnostics. The transit exercise presented here indicates that *in vivo* use of commercially available technology to screen dozens to hundreds of sites in a surgical theater will require a significant increase in data acquisition rate beyond the current 90 seconds used for the transit experiments and even the 10 seconds required when using the hand-held probe.

The preliminary data presented in this project confirms the work of multiple other groups documenting that near-infrared laser Raman spectroscopy can identify spectral signatures for healthy and neoplastic breast tissue^55–57^. To utilize the diagnostic information that is widely distributed across multiple Raman peaks, factor analysis has taken a prominent role in breast cancer diagnostics over the last decade and has been of considerable utility in this study^11,58–63^. For example, Brozek-Pluska and coworkers employ 532 nm confocal Raman spectroscopy for the characterization of malignant and healthy tissue using paraffin-fixed thin sections with a specificity that makes it possible to identify subtle shifts in lipid composition^18^. Haka and her colleagues have developed basis spectra representing the major biological components of breast tissue, fit the bases to spectra collected from breast tissue, and then used fit coefficients to discriminate between healthy and malignant tissues^19^. Sathyavathi and coworkers have discriminated benign from malignant breast lesions by measuring micro-calcifications via the calcium carbonate Raman signature^20^. Our data support the findings of these investigations that laser Raman spectroscopy combined with PCA-LDA analytic techniques can identify significant differences between cancerous and healthy tissue.

In our experiments, we collected malignant spectra from invasive ductal carcinoma of the breast (IDC). While IDC represents the most common type of breast cancer, invasive lobular carcinoma (ILC) represents a significant minority. ILC has a distinct morphology, and is typically subtly infiltrative and can be difficult to detect on routine H&E-stained pathology slides. In future work, we plan to evaluate the performance of Raman spectroscopy for the detection of ILC and other more uncommon types of breast cancer.

We expect that, with the increasing world-wide effort to introduce continued adoption of Raman technology for cancer diagnostics^16^, the exceptional specificity of the technique will identify relatively small, but highly consistent shifts in selected bands reflecting the appearance of pre-cancerous lesions^17,64^, degree of cell transformation^65^, treatment response (chemotherapy, immunotherapy, or radiation)^66,67^, and fatty acid^32^. Raman technology is unlikely to replace standard postoperative pathologic evaluation of breast cancer specimens. Rather, we envision a scenario where Raman offers the breast surgeon a method for rapid and accurate statistical assessment of positive margins at the time of surgery. Such a method would spare the patient additional surgery, anxiety, morbidity and healthcare expenditure.

## Methods

### Raman instrumentation

We evaluated two commercial Raman systems. Both systems operate in the infrared, one using a 1024 nm laser excitation source and the other operating at 785 nm. Both wavelengths are known to be capable of interrogating biological systems without damaging target material. The 1064 nm systems probe more deeply into tissue than a 785 nm device and often generate significantly less fluorescence than shorter, more energetic laser wavelengths. That is a significant advantage since fluorescence can easily mask the weaker Raman signal. Unfortunately, systems operating at 1064 nm are significantly more expensive and usually exhibit a more limited spectral bandwidth and diminished spectral resolution. The two systems evaluated were the i-Raman Ex 1064 nm and i-Raman Plus 785 nm, both manufactured and distributed commercially by B&W Tek (Newark, DE). Both systems can be operated in microscopic or hand-held probe modes. For initial evaluation, we employed the systems in microscopic mode and selected laser exposure times so that total laser exposure (laser excitation power x collection time) would equal 9 × 10^3^ mW-seconds for both systems. Our first evaluation focused on the impact of tissue fluorescence on Raman signatures. Historically, the fluorescence response to laser excitation can be as much as three orders of magnitude greater than the Raman scattering signal. Evaluation requires analyzing the raw spectra generated by each system.

The i-Raman Plus system uses a high quantum efficiency 2048-pixel CCD array detector, with a spectral resolution of 4.5 cm^−1^ and a spectral coverage range of 150–2250 cm^−1^. The detector cooled temperature is −2 °C with a typical dynamic range of 50,000:1 and integration time ranging from 100 milliseconds − 30 minutes. The effective pixel size is 14 μm × 9 μm. The i-Raman EX system uses a thermoelectrically cooled, 512-pixel InGaAs array detector with coverage range of 100–2500 cm^−1^ and resolution of 9.5 cm^−1^. The detector cooling temperature is −20 °C with dynamic range greater than 100,000:1 and effective pixel size of 25 μm x 25 μm. Integration time can range from 200 μs to greater than 30 minutes.

In each device the spectrometer housing connects via fiber optic cables to the BAC102 Raman Trigger Probe. The probe has a spot size of 50–85 um. Table 4 summarizes the physical differences in the sensors for the two systems. Since the 1064 nm system is equipped with a 512 pixel sensor, while the 785 nm system employs a 2048 pixel detector, the effective response for the 1064 nm system covers a spectral bandwidth of only ~2253 cm^−1^ from 247.1–2499.69 cm^−1^, spans 428 pixels, and provides 5.07 cm-1resolution (inter-pixel distance) at 1600 cm^−1^. The 785 nm system has an effective bandwidth of ~3026 cm^−1^ between 174.79 and 3201.06 cm^−1^, spans 1804 pixels and produces a 1.78 cm^−1^resolution limit at 1600 cm^−1^.

### Tissue preparation and histology

Tissue samples were collected following surgical resection under IRB protocol at City of Hope (COH) in Duarte, California (VJ, LL, and YF, COH IRB #16317, renewed 07/23/2019) and only after patients provided informed consent. Following resection, tissue samples were immediately frozen and stored at −80 °C for post-operative Raman evaluation. For spectral analysis samples were thawed ~5–10 minutes before data collection. The pathologist on our team (DS) identified three breast tissue zones on each sample by simple, macroscopic visual inspection: healthy, tumor, and the tissue that appeared between these two sites was deemed the transition zone. All excised tumors in our study were invasive ductal carcinomas of the breast. Once spectral data were obtained, standard hematoxylin and eosin (H&E) glass slides were prepared. These slides were digitally scanned at 20X magnification using a Ventana iScan HT slide scanner (Roche Holding AG, Basel, Switzerland). The resulting whole slide images were assessed using the QuPath open source imaging application (Queen’s University Belfast, Belfast Northern Ireland, UK) to determine the microscopic heterogeneity of cancerous and healthy cells at target sites in the three macroscopic tissue zones.

Clearly, perfect co-registration between the standard H&E 2-D slide and 3-D sample is not achievable for several reasons. First, a certain amount of tissue is discarded in the process of “facing up” the paraffin embedded tissue block to produce a square surface for microtome sectioning, thus introducing localization uncertainty in the z plane. In addition, slight differences in camera angles and specimen rotation in 3-D space during sectioning add geometric positioning and imaging uncertainty in the XY-plane. Following co-registration of the visible light microscopic images with our spectral target position grid, we assign 1 mm “best guess” error bars for positioning accuracy.

### Raman acquisition and data processing

Prior to data collection, calibration spectra were obtained using Teflon standard targets. During data acquisition, BWSpec, the software integral to the i-Raman Plus and i-Raman EX systems, applies a baseline subtraction for ambient noise, and filters cosmic ray anomalies. The data were then corrected for fluorescence using MATLAB’s msbackadj.m function. The function iteratively estimates the spectral baseline using shifted windows and regression with a spline approximation, then subtracts the predicted fluorescence contribution from the signal. The final spectrum is normalized to the area under the curve between 400 and 1800 cm^−1^ for the1064 nm system, and between 400 and 3200 cm^−1^ for the 785 nm system.

To implement a real-time machine learning system on a local data set that is sufficiently rigorous to identify tumor spectral signatures in a broader population of samples, we elected to minimize the number of potential input variables (428 in the case of the 1064 nm system and 1804 for the 785 nm device). First, we calculate the 95% confidence interval for spectra from healthy and cancerous tissue and identify the regions that maximize the area between the confidence interval boundaries. We also characterize and exclude from classification the spectral regions containing Raman activity originating from the dyes used to provide tissue landmarks during surgical excision.

### Multivariate analysis

Once the discriminating spectral bands were identified and the data were mean-centered, two multivariate techniques, Principal Component Analysis (PCA)^60^ and Linear Discriminant Analysis (LDA)^68^ are employed in the experiments reported here for feature extraction and variable input reduction (PCA) and classification (LDA). PCA, also known as the Karhunen-Loéve or Hotelling transform, extracts significant information from a data set by identifying linear combinations of raw variables accounting for maximum variance in the data set. PCA identifies correlations or covariance between multiple variables and calculates a new variable, the first principal component, which accounts for as much variance in the data as possible. The process continues generating successive factors that account for decreasing fractions of the total variance. The method is robust to moderate amounts of noise since the covariance matrix is an average over many input vectors and the noise is uncorrelated from one data vector to the next. In most experiments, a practical balance between data compression and classification accuracy can be achieved by selecting eigenvectors that account for 75–95% of the eigenvalues. The new set of PCA factors encodes in compressed format the significant information content of the original data set. PCA is an unsupervised classifier, meaning it does not use a priori classification designations.

LDA, also known as the Fisher discriminant, is a classification method that assumes different classes generate data based on differing Gaussian distributions. To train a classifier, the fitting function estimates the parameters of a Gaussian distribution for each class. To predict the classes of new data, the trained classifier finds the class with the smallest misclassification cost. Cross-validation using a leave on out format is employed for seamless training and testing of a data set. Both PCA and LDA are linear transformation techniques commonly used for multivariate data analysis. PCA in combination with LDA has been shown to improve Raman spectral classification sensitivity and specificity^14^. These were employed to analyze the spectra and reliably distinguish malignant from benign tissue.

## Supplementary information

Supplementary Figure S1
