# Supplementary material for: Raman Spectroscopy for Rapid Evaluation of Surgical Margins during Breast Cancer Lumpectomy
Source: Sci Rep. 2019 Oct 10;9:14639. doi: 10.1038/s41598-019-51112-0 (PMC6787043; doi:10.1038/s41598-019-51112-0)
Supplement: Supplementary file 1 — Supplementary Figure S1 [file 41598_2019_51112_MOESM1_ESM.docx]

# Supplementary Figure

**Figure S1**


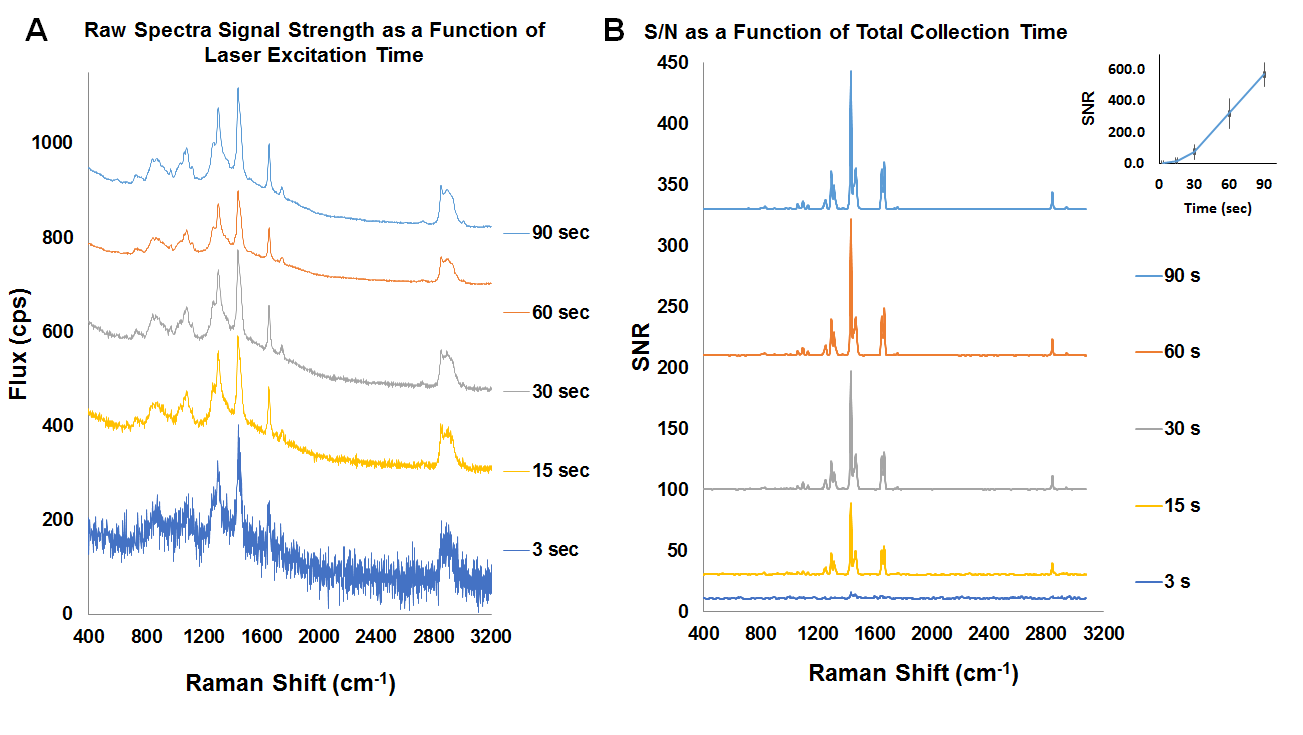


**Figure S1. Optimization of spectral collection parameters.**

To estimate how rapidly we might proceed with multiple scans of fragile tissue samples, at the beginning of the experiment we evaluated the signal-to-noise characteristics of the 785 nm system for collection times ranging from 3 to 90 seconds (see supplementary Figure S1). The signal-to-noise ratio (SNR) of a spectral signature is most often defined as the ratio of the variance of the signal relative to the variance of the noise,

$SNR= \frac{\sigma_{signal}^{2}}{\sigma_{noise}^{2}}$ (1)

where ***σ*** denotes the standard deviation. To select sample exposure time, five spectra were acquired with 100 mW power for 3, 15, 30, 60, and 90 seconds from a region of healthy tissue (labelled 1-5 in Fig. 6A and 6B). Figure S1 shows the resulting spectra acquired for each of these exposure times in two formats. Fig. S1A depicts the raw spectra in counts per second (cps). Fig. S1B, depicts the SNR for each spectrum as a centered running estimate of signal variance (n=11 spectral bins) is divided by the noise variance estimated using the feature-barren region from 1900 to 2600 cm^-1^. The inset shows that the change in SNR as a function of collection time ranges from a ratio of 2.9 for 3 seconds laser exposure to a maximum ratio of 639 for 90 seconds. To ensure detection of subtle shifts in spectral signatures while transiting tumor margins, we chose the conservative 90 seconds data acquisition time.
